# Can mindfulness have long-term impact on patients with fibromyalgia? A two-year prospective follow-up study of a mindfulness-based intervention

**DOI:** 10.1007/s00296-024-05778-z

**Published:** 2025-01-07

**Authors:** Heidi A. Zangi, Trond Haugmark, Sella Aarrestad Provan

**Affiliations:** 1 Health Service Research and Innovation Unit, REMEDY Center for Treatment of Rheumatic and Musculoskeletal Diseases, Diakonhjemmet Hospital, Box 23, Vinderen, Oslo, 0319 Norway; 2 Faculty of Health Science, Institute of Health, VID Specialized University, Oslo, Norway; 3 Veidekke ASA, Oslo, Norway; 4 Clinic for Rheumatology, Outpatient Clinic and Research, REMEDY Center for Treatment of Rheumatic and Musculoskeletal Diseases, Diakonhjemmet Hospital, Oslo, Norway; 5 Faculty of Public Health, Inland Norway University of Applied Sciences, Oslo, Norway

**Keywords:** Fibromyalgia, Mindfulness, Prospective studies, Long-term follow-up

## Abstract

To examine changes in symptoms and health status in patients with fibromyalgia (FM) 24 months after participating in the mindfulness-based group-program, the Vitality Training (VTP), followed by physical exercise counselling. Seventy-six participants, mean age (range) 43 (26–52), females 69 (91%), diagnosed with FM according to the ACR 2011-criteria received the VTP in a previous randomised controlled trial. Control group participants could receive the VTP after a 12-month observation period, therefore only data from the intervention group were analysed in the present study. Self-reported data were collected electronically at baseline, 3, 12 and 24 months. Outcomes were patient global impression of change (PGIC), FM-severity, i.e. widespread pain (WPI) and symptom severity (SSS), pain, fatigue, sleep quality, psychological distress, motivation and barriers for physical activity, mindfulness and work participation. Trends across time-points were analysed using mixed models for repeated measurements. At 24-months, 48 (56.5%) participants responded, 94% female, median (range) age 46 (28–54), symptom duration 12 (5–33) years. Seven participants reported much/very much better on the PGIC; 21 (44%) reported no change/minimal improvement. Improvements were observed in WPI (-1.9, ES 0.4), SSS (-1.2, ES 0.6), fatigue (-0.8, *p* =.014) and self-efficacy for physical activity (1.4, ES 0.4). There was a significant trend of reduced WPI, SSS, pain and fatigue across the four time-points, but no additional improvements from 12 to 24-month follow-up. Participants who had completed the VTP demonstrated small to moderate improvements in symptom burden and FM-severity from baseline to 24-month follow-up. Trial registration number: ISRCTN96836577 10.1186/ISRCTN96836577, prospectively registered 12.07.2016.

## Introduction

People with Fibromyalgia (FM) can suffer from a high symptom burden, including pain, fatigue, sleep disturbances, and other somatic and functional symptoms [1]. Due to a lack of effective treatments, patients largely must self-manage their condition. The EULAR evidence-based recommendations for the management of FM state that optimal management should comprise prompt diagnosis, patient education, and non-pharmacological treatments including physical exercise and behavioural interventions [2]. Several studies support integrating mindfulness into healthcare as part of selfcare, disease management and prevention of stress-related physical symptoms for people with chronic conditions [3]. Systematic reviews of mindfulness-based interventions for people with FM have shown small to moderate beneficial health effects [4, 5, 6]. The effects of mindfulness may only be fully realised when it is integrated into the lives of patients, but there is a lack of studies that include long-term follow-up of mindfulness interventions.

We conducted a two-armed parallel randomised controlled trial (RCT) to evaluate effects of a 10-session mindfulness-based group intervention, the Vitality Training Program (VTP), followed by individual physical exercise counselling for people recently diagnosed with FM [7]. The wait-list control group could follow their usual treatment. At the 12-month follow-up there were no significant between-group effects in patients’ perceived changes in health status or FM symptoms. However, there was a statistically significant increase in tendency to be mindful, and a tendency towards significant improvement in pain in the intervention group compared to the control group [7]. The present study aimed to evaluate if there were any long-term improvements in symptom burden in patients with FM 24 months after participating in the VTP.

## Methods

**Fig. 1:**
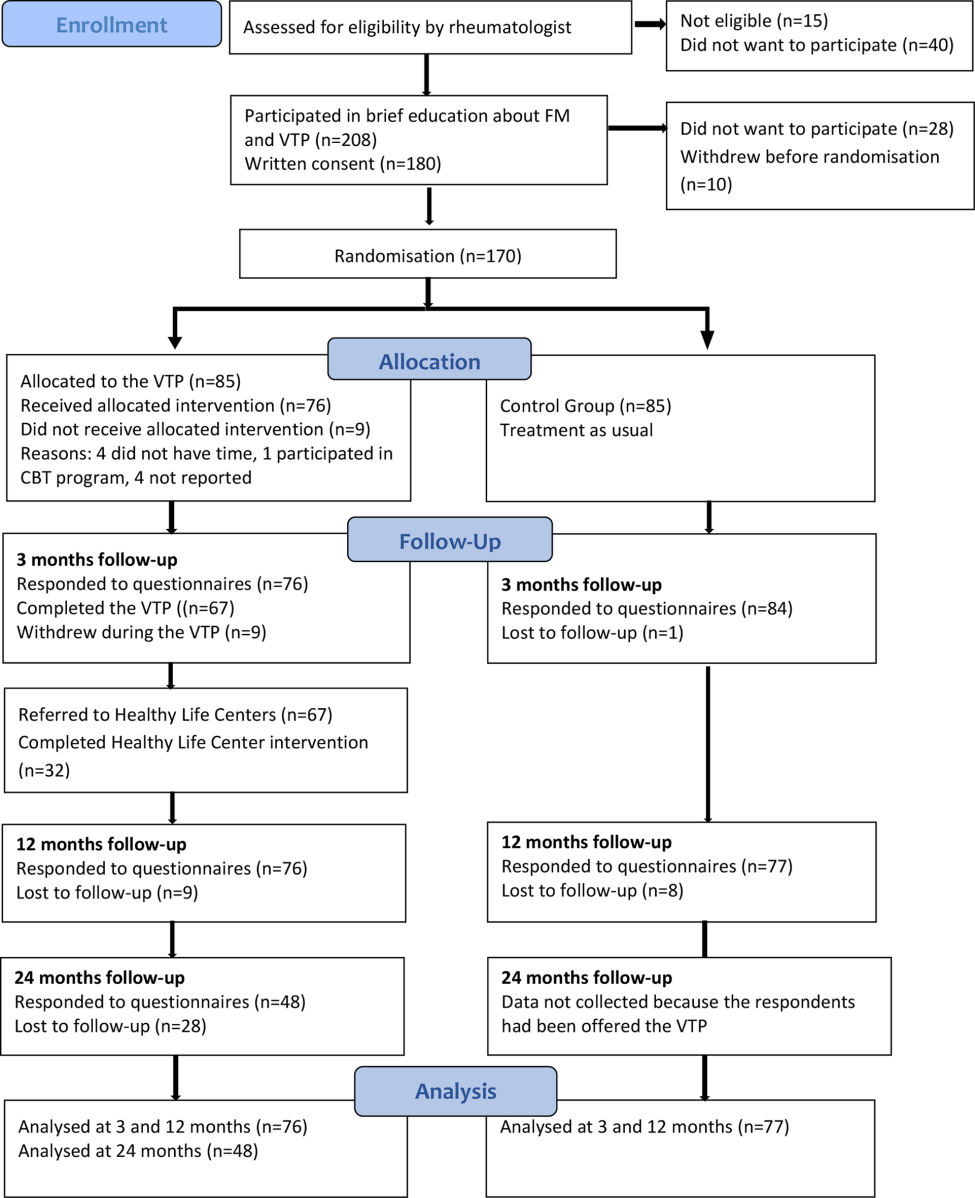
Flow chart of participants throughout the study

The RCT was conducted during 2017 to 2020 following a published study protocol [8]. Individuals with widespread pain of three or more months’ duration were referred from general practitioners to rheumatologists in a specialist health care clinic for diagnostic clarification and assessment of co-morbidities. Patients were eligible for inclusion if they were aged between 20 and 50 years and were diagnosed with FM by a rheumatologist according to the American College of Rheumatology 2011-criteria [9]. Exclusion criteria were being diagnosed with inflammatory arthritis, being out of work for more than the two last years and being unable to understand Norwegian. All eligible participants received a 3-hours patient education program and information about the study before they signed informed consent. Those who accepted to participate were randomised by a blinded statistician according to an electronically generated randomisation list to intervention (*n* = 85) and control (*n* = 85) groups. A full description of the included participants and study procedures in the RCT have been reported in a previously published paper [7]. Figure 1 illustrates the flow of patients through the study.

The VTP was delivered in groups of 7 to 12 participants in weekly 4-hour sessions over 10 weeks. The mindfulness training comprised shorter meditation exercises and integration of attitudes such as being non-judgemental, non-striving and accepting. In addition, the participants were invited to explore topics like *If my body could talk*/*Values-what is important to me/What do I need/Bad conscience/Anger/Joy/Resources*,* potentials and choices* by creative and interactive methods. The group facilitators provided individual counselling within the group setting to help participants identify their personal values and resources. A detailed description of the program can be found in previous publications [7, 10].

After completion of the VTP, the participants were referred to individual physical activity counselling by a physiotherapist at a community-based Healthy Life Centre that typically offer a daytime program over 12 weeks based on motivational interviewing. The aim was to help participants setting tailored goals, identifying and overcoming barriers to physical activity, and guiding them into exercises that they could continue after the 12-week period.

Self-reported questionnaires comprising baseline demographics and all outcome measures were collected electronically before randomisation (baseline), after the VTP (3 months) and at 12 and 24 months. Outcomes were FM disease severity measured by the Polysymptomatic Distress Scale (PSD), comprising Widespread Pain Index (WPI) and Symptom Severity Scale (SSS) [11]. The PSD is scored from 0 to 31, scores 4 to 7 indicate mild disease, 8 to 11 moderate disease and 12 or above severe disease [11]. Other outcomes were pain, fatigue, and sleep quality measured by Numerical Rating Scales, psychological distress measured by General Health Questionnaire-12 [12], Exercise Beliefs and Exercise Habits [13]; and tendency to be mindful measured by Five Facet Mindfulness Questionnaire [14]. Participants perceived change in health status from baseline was measured by Patient Global Impression of Change (PGIC, 1–7), scores 6 and 7 indicated “feeling much or very much better” [15].

All participants retained their original allocated groups throughout the study. The participants in the control group could receive the VTP after the 12-month data collection. Therefore, only data from the intervention group were analysed at 24-month follow-up. The distribution of scale variables was visually examined and the means calculated for variables with a normal distribution. Missing values in single items were imputed by the last value carried forward. The mean changes from baseline to 24 months were analysed by paired sample t-tests and effect sizes calculated by Cohen’s d [16]. The trend for change in symptom severity over time was estimated by random effect mixed models for repeated analyses where each outcome variable was the dependent variable in separate models. Time was entered as an independent variable in models that were also adjusted for age. Data were analysed using SPSS version 29.0.

## Results

**Fig. 2:**
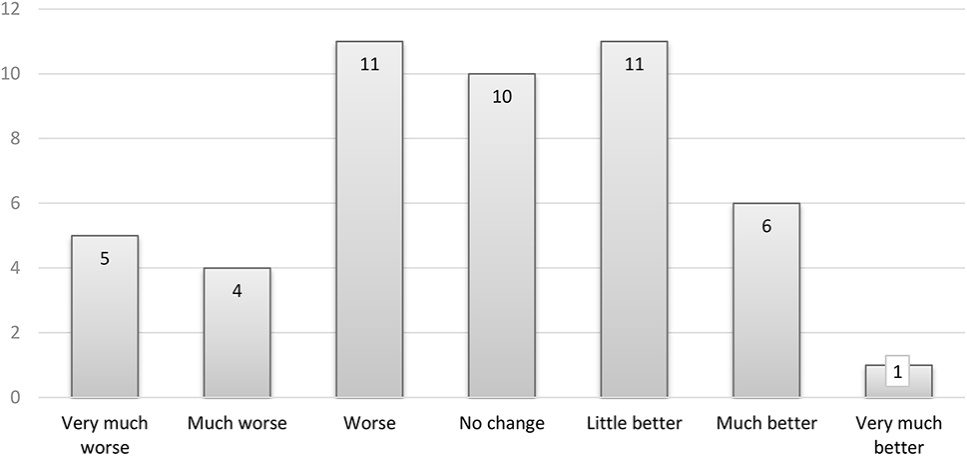
Patient global impression of change (PGIC, 1 - 7)

Slightly over half of the intervention group participants responded to the questionnaires at the 24-month follow-up, *n* = 48 (56.5%). Of these respondents, 94% were female, with a median (range) age 46 (28 to 54) and symptom duration 12 (5 to 33) years. Baseline characteristics for all participants who started in the interventions group (*n* = 76) and for those who responded at 24 months (*n* = 48) are shown in Table 1. At 24 months, only seven participants reported feeling much or very much better on the PGIC, while 21 (44%) reported no change/little better, and 20 (42%) participants perceived their health status as worse (Fig. 2). Among those who responded at 24 months, 58% were employed compared to 73% at baseline.

**Table 1:** Changes from baseline to 24 months follow-up and across all time-points

Outcomes^*^	Baseline intervention group^*^(n = 76) mean (SD)	Baseline respondents 24 months (*n* = 48) mean (SD)	24- month follow-up (*n* = 48) mean (SD)	Mean Change (CI) from baseline to 24 months in 48 respondents	*P*	Effect size (Cohen’s d)	Trend across all time-points	*P* change across time-points
Widespread pain index (WPI, 0–19)	*12.0 (3.7)*	12.8 (3.8)	10.9 (4.6)	-1.9 (-0.6, -3.2)	0.006	0.4	-0.9 (-1.3, -0.5)	< 0.001
Symptom Severity Scale (SSS, 0–12)	*8.4 (2.0)*	8.5 (1.8)	7.3 (2.2)	-1.2 (-0.6, -1.8)	< 0.001	0.6	-0.5 (-0.7, -0.2)	0.002
Pain last week (NRS^1^, 0–10, 0 = no pain)	*6.7 (1.7)*	6.8 (1.5)	6.2 (1.7)	− 0.6 (-0.01, -1.1)	0.048	0.3	-0.2 (-0.4, -0.0)	0.01
Fatigue last week (NRS^1^, 0–10, 0 = no fatigue)	*7.4 (2.0)*	7.6 (1.9)	6.8 (2.1)	-0.8 (-0.2, -1.4)	0.014	0.4	-0.3 (-0.5, -0.1)	0.01
Sleep last week (NRS^1^, 0–10, 0 = no sleep problems)	*6.9 (2.3)*	6.8 (2.1)	6.6 (2.1)	− 0.3 (-0.3, 0.8)	0.402	0.1	-0.1 (-0.3, 0.1)	0.57
Psychological distress (GHQ-12^2^ sum), 0–36, 0 = no distress	*16.1 (6.4)*	16.2 (6.4)	14.9 (6.9)	-1.3 (-1.1, 3.6)	0.293	0.2	-0.1 (-0.8, 0.5)	0.68
Physical activity^3^, 0–15, 0 = inactive	*3.0 (2.5)*	3.1 (2.6)	2.1 (2.2)	-1.0 (-0.2, 2.2)	0.095	0.3	0.01 (-0.2, 0.2)	0.95
Self-Efficacy for physical activity^4^ 4–20, 20 = high SE	*12.0 (3.0)*	12.0 (3.1)	13.4 (3.6)	1.4 (2.4, 0.3)	0.012	0.4	0.44 (0.2, 0.7)	0.002
Barriers for physical activity^4^ 3–15, 15 = low barriers	*12.0 (2.4)*	12.4 (2.2)	12.1 (2.4)	-0.3 (-0.3, 0.9)	0.320	0.2	0.0 (-0.2, 0.1)	0.68
Benefits of physical activity^4^, 5–25, 25 = high benefits	*20.7 (2.8)*	20.5 (3.1)	20.0 (3.1)	-0.5 (-1.3, 1.3)	0.136	0.2	-0.1 (-0.3, 0.1)	0.39
Impact of physical activity^4^ 8–40, 40 = high positive impact	*28.7 (4.9)*	28.8 (4.7)	28.9 (6.5)	0.01 (-1.3, 1.3)	0.974	0.01	0.1 (-0.3, 0.4)	0.64
Mindfulness^5^, sum 39–195, 195 = high tendency to be mindful	*119 (16.3)*	117 (15.5)	124 (16.4)	6.9 (11.3, 1.5)	0.012	0.3	2.0 (0.7, 3.3)	0.003

*Reference: Haugmark T et al. BMJ Open 2021, ref. #7

^1^NRS = Numerical Rating Scale, ^2^GHQ-12 = General Health Questionnaire-12, ^3^Physical activity measured by three questions from the Norwegian population-based “North-Trøndelag Health Study”, ^4^Exercise Beliefs and Exercise Habits, ^5^Five Facet Mindfulness Questionnaire (FFMQ)

SD = Standard Deviation, CI = Confidence Interval

**Fig. 3:**
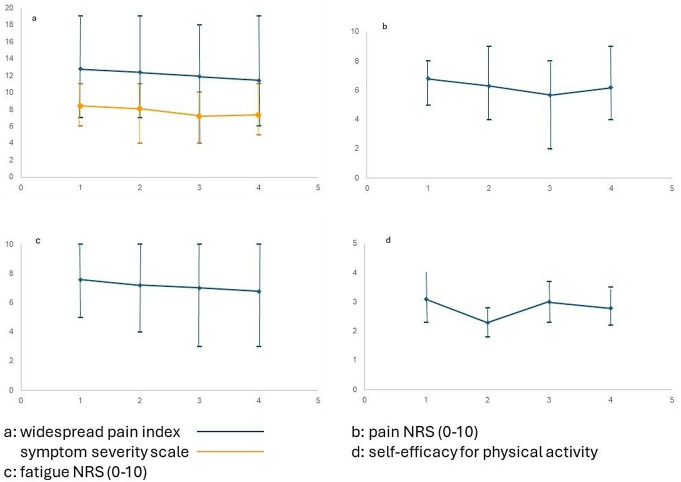
**a**-**d**: Changes across the four time-points for variables with a significant trend

There were small to moderate improvements from baseline to 24 months in the WPI and SSS (Table 1). At baseline, all participants reported ‘severe or very severe disease’ (i.e. PSD ≥ 12). A smaller proportion of the participants scored ‘very severe’ at 24 months (37%) compared to baseline (54%). One participant reported only mild disease. There were significant trends of reduced WPI, SSS, pain and fatigue across all four time-points (Fig. 3a-d). Self-efficacy for physical activity and mindfulness showed increased trends (Table 1). However, there were no significant improvements between the 12- and 24-month follow-up (data not shown).

## Discussion

We investigated long-term changes in symptom burden in patients with FM who had participated in a mindfulness-based group-program, the VTP, followed by physical activity counselling. Just over half of the participants in the intervention group responded to the electronic self-reported questionnaires at the 24-month follow-up. Statistically significant improvements were observed in FM severity, pain, fatigue, and self-efficacy for physical activity compared to baseline, but there were no significant changes between 12 and 24-month follow-up. There was also a sustained improvement in the tendency to be mindful. These changes were of small to moderate magnitude and were not reflected in participants’ self-perceived improvement in health status.

FM is known to be a fluctuating condition with symptoms that vary over time. It is possible for some patients, particularly those who are diagnosed early, to recover from the diagnosis [17]. To increase the likelihood of decreasing symptom burden and promoting recovery, we included participants who had recently been diagnosed with FM. However, only one participant demonstrated recovery based on the PSD score. Most participants reported experiencing symptoms for several years, suggesting that there may still be a delay in diagnosing individuals with FM. Our findings are consistent with a large two-year observational study in the United States involving 226 FM patients, which showed that the majority of patients still met the FM diagnosis criteria after two years [17]. However, our study demonstrated that there was a displacement of participants from ‘*very severe’* to ‘*severe’* disease, indicating a slight improvement in symptom burden.

Previous research has shown that the VTP improved psychological distress, fatigue, and self-efficacy in patients with inflammatory arthritis [10] and chronic pain [18]. Although only a few participants reported better health status in the present study, the small to moderate long-term improvements in pain and other symptoms may be attributed to the participants having integrated new attitudes and practices into their lives. However, there were no significant improvements at 24 months compared to 12-month data, suggesting that regression to the mean may be an explanation of the observed change.

From a clinical perspective, a critical question is how we can strengthen patients’ ability to manage their daily lives and maintain their health with their fluctuating FM condition. The VTP aims to increase participants’ awareness of their internal and external resources and strengthen their ability to cope with stress through mindfulness techniques. The group-counselling encourages a non-judgemental, non-striving and friendly attitude towards oneself and helps participants reduce their struggle to control pain and promote acceptance, which are the core values in mindfulness-based interventions [5]. A qualitative sub-study involving six FM patients who had completed the VTP found that the program had helped participants adopt a more accepting attitude towards themselves, cultivate belief in their coping abilities, and shift their attention from a disease perspective to a health-promoting perspective [19]. Participants described continuing this process after completing the program [19]. However, these changes could not be captured with the quantitative measures we applied in our study. Although we cannot generalise from a small qualitative study, it indicates that other coping measures might have been more relevant to capture changes.

Physical activity should be recommended to all patients with FM [2]. In our study, participants were offered low-threshold physical activity counselling over a 12-week period to guide them into activities that they could easily continue after the 12-month period. However, less than 50% of the participants completed the physical activity intervention. Therefore, it was not possible to determine whether this had influenced the participants’ health status. This issue has been discussed in a previous publication [7]. There was an increase in participants’ self-efficacy for physical activity over the follow-up time-points, but this was not reflected in patients reporting being physically active.

Most participants reported no change in their perceived health status over the two-year period. The validity of PGIC as a measure for long-term follow-up in FM can be questioned. In a real-life assessment of the validity of PGIC in FM, longer duration of follow-up was identified as a predictor of lower patient impression of change. Patient reporting may be influenced by recall bias, reinterpretation of symptom meanings, and other life events that have impacted their health [15].

Only a few intervention studies have followed FM patients over two years. A two-year follow-up cohort study on recently diagnosed FM patients demonstrated that patients with fewer physical limitations experienced more health improvements if they were working and had a positive attitude towards participating in a healthcare interventions [20]. In our study, all patients had participated in a healthcare intervention. However, we did not find any associations between work status and improvement in health.

One major limitation in our study is that we could not compare the intervention and control groups at the 24-month follow-up. Therefore, we cannot confirm whether the moderate changes observed were due to natural fluctuations in symptoms or if participants had integrated new practices that had influenced their symptom perception. Additionally, just over half of the patients who participated in the intervention responded at the 24-month follow-up. It is possible that these participants had a more positive attitude towards the intervention and thus practiced what they learned. However, we did not detect significant differences at baseline between those who responded at 24 months, and those who did not. Although ethical reasons may justify offering the control-group participants the intervention after a one-year follow-up, we still recommend more controlled trials with a two-year follow-up to further explore the long-term impact of mindfulness-based interventions. Furthermore, it is a need to explore how interventions can be adapted to individual patients’ needs and what characterizes patients who benefit from mindfulness-based interventions.

In this 24-month follow-up study, individuals with FM who had participated in the VTP followed by physical activity counselling demonstrated small to moderate improvements in patient-reported symptoms and FM severity. However, no improvements were observed when compared to the 12-month follow-up. The improvements were not reflected in patients’ self-perceived health status. Because we were unable to compare with a control group at 24 months, changes in symptoms may be attributed to a shift patients’ perceptions of their disease and coping abilities, as well as natural fluctuations in symptoms or regression to mean.

## Declarations

### Open data sharing note

Data are available on request to the corresponding author.

### Note about related congress abstract publication

Zangi HA, Haugmark T, Provan S Aa, Hagen KB. High symptom burden and limited changes in fibromyalgia patients’ health status two years after participation in a multicomponent rehabilitation programme. DOI: 10.1136/annrheumdis-2022-eular.4532.
